# Puumala Virus Infection with Acute Disseminated Encephalomyelitis and Multiorgan Failure

**DOI:** 10.3201/eid0905.020405

**Published:** 2003-05

**Authors:** Robert Krause, Stephen Aberle, Renate Haberl, Florian Daxböck, Christoph Wenisch

**Affiliations:** * Karl-Franzens University Graz, Graz, Austria; † University of Vienna, Vienna, Austria

**To the Editor:** Hantaviruses, which belong to the genus *Hantavirus*, family *Bunyaviridae*, are human pathogens that are prevalent worldwide (*1*). More than 16 different genotypes or serotypes have been identified (e.g., Puumala, Hantaan, Dobrava-Belgrade, Seoul, Sin Nombre). In western and central Europe, the predominant serotype is Puumala, which causes nephropathia epidemica. Puumala virus (PUUV) is spread by rodents and is transmitted to humans by inhalation or ingestion of food contaminated with rodent excreta (*2*). Nephropathia epidemica is endemic in western Russia, Finland, Sweden, France, Belgium, Germany, and former Yugoslavia. Reports of serologically verified nephropathia epidemica cases have also been published from Denmark, Norway, the Netherlands, and Austria (*3*). In Austria, the risk for infection seems to be restricted to special areas in Styria and Carinthia where *Clethrionomys glareolus*, the reservoir of PUUV in Austria, is endemic. The seroprevalence in Finland is 5% and 1.8% in Austria (*4*). The most common symptoms of nephropathia epidemica are fever, nausea, vomiting, headache, stomachache, back pain, tenderness in the kidney area, diarrhea or constipation, and red throat (*5*). PUUV infection may also lead to neurologic symptoms including meningoencephalitis, polyradiculitis, seizures, cerebral hemorrhage, urinary bladder paralysis, and hypopituitarism (*6*,*7*).

Our patient, a 43-year-old previously healthy man, had a temperature of 39°C and acute abdominal pain. Two days after the symptoms began, he was admitted to a regional hospital where acute renal failure and disseminated intravascular coagulation developed in the next 2 days. The patient was transferred to the Department of Medicine, Karl-Franzens University Graz, for intensive care. The patient worked in a factory, and he hunted in his spare time. A few days before his illness began, he had cleaned up his hut in the forest.

On admission to the intensive care unit, physical examination showed abdominal guarding and a body temperature of 39.2°C. Laboratory tests showed thrombocytes 36 G/L (140–440 G/L), creatinine 3.6 mg/dL (0.6–1.3 mg/dL), urea 132 mg/dL (10–45 mg/dL), D-dimere 1,558 µg/L (<200 µg/L), ATIII 67% (>75%), c-reactive protein (CRP) 237 mg/L (<9mg/L), lactate dehydrogenase (LDH) 322 U/L, and slightly elevated liver enzymes. Computer tomography (CT) of the thorax showed bilateral opacities in the lungs and pleural effusion. In the CT of the abdomen, a thickened wall of the colon ascendens, an enlarged caecum, slightly enlarged kidneys, approximately 500 mL of ascites, and enhancement of the peritoneum were found. Gastroscopy and colonoscopy results were normal. In the ascites, protein of 3.1 g/dL and 1,000 cells/L with 73% neutrophils were detected. A few hours after admission to the intensive care unit, the patient’s level of consciousness started to deteriorate, and respiratory failure and circulatory insufficiency with a blood pressure of 78/50 developed. He was intubated and ventilated, received catecholamines, and was empirically treated with meropenem and clarithromycin adjusted to renal function. Liquor examination showed elevated lactate (2.7 mmol/L; normal range 2.1 mmol/L) and elevated protein (67 mg/dL; normal range 45 mg/dL). Detailed cerebral spinal fluid testing did not show additional information. Despite antibiotic therapy, abdominal tenderness, organ functions, and laboratory test results worsened. Four days later antibiotic therapy was changed to ciprofloxacin and metronidazole adjusted to renal function and the patient was hemodialized. Because of increasing ascites, ileus, and raising CRP (from 216 to 391 mg/L) in the next 3 days, explorative laparotomy was performed, but no focus of infection could be found. One day after surgery, meningism and hyperreflexia developed. A brain CT showed wide areas of hypodensity bilateral in the white matter partially involving the cortex. Magnetic resonance imaging (MRI) showed bilateral areas of increased signal intensity located in the parietooccipital region extending to the frontal, temporal, and pons regions and associated with cerebral edema. The lesions predominately affected the white matter but, particularly in the occipital region, also involved the cortex. Because of the patient’s history and his recent activities in his forest hut, serum samples were investigated for antibodies against PUUV*, Leptospira* sp., *Ehrlichia* sp., *Borrelia* sp., *Francisella tularensis*, *Bartonella henselae*, and *Coxiella burnetii*. PUUV antibodies were found to be positive (highest titers: immunoglobulin (Ig) M 1:64, IgG 1:8000) in an immunofluorescence test (Progen, Heidelberg, Germany) and an immunoassay (Mikrogen, Martiensried, Germany).

The patient further received catecholamines, hemodialysis, and mechanical ventilation. In the week after surgery, he improved clinically, and catecholamines, hemodialysis, and mechanical ventilation were stopped 15 days after initiation. One week later, a second brain MRI showed resolving abnormalities. Four weeks after admission to the intensive care unit, the patient left the hospital in good condition. Two months later, MRI of the brain was normal, and the patient was well at an 18-month follow-up.

A few reports of hantavirus infection with cerebral involvement have been published. Recently, a patient with acute disseminated encephalomyelitis following nephropathia epidemica was reported (*2*). Whereas this patient had acute renal failure and acute disseminated encephalomyelitis, our patient suffered from multiorgan failure with respiratory, circulatory, and renal insufficiency, paralytic ileus, disseminated intravascular coagulation, and acute disseminated encephalomyelitis. In addition, in our patient, the disseminated encephalomyelitis involved parietooccipital, temporal, and frontal regions of the brain and also reached the brain stem. Other causes of acute disseminated encephalomyelitis such as multiple sclerosis, encephalitis caused by other infectious agents, uremic encephalitis, and hypertensive encephalitis could be ruled out.

In our patient, abdominal pain, ileus, thickened wall of the colon, and enlargement of the caecum mimicked acute abdomen, which has also been reported in two other cases of hantavirus infection (*8*). Usually hantaviruses are transmitted by inhalation of virus-containing particles originating from rodents urine, droppings, and saliva. Therefore, transmission can occur at any place that infected rodents have infested (*9*). In our patient, the probable source of infection was his housecleaning activities in his hut a few days before his illness. Since this hut served as a storage facility and was rarely entered, it was occupied by rodents.

In summary, PUUV infection should be considered in the differential diagnosis of multiorgan failure and acute disseminated encephalomyelitis, especially in patients from PUUV-endemic areas and typical history.
